# Insulin-Like Growth Factor Binding Protein 5—A Probable Target of Kidney Renal Papillary Renal Cell Carcinoma

**DOI:** 10.1155/2019/3210324

**Published:** 2019-11-11

**Authors:** Shuqiang Wang, Quan Hong, Xiaodong Geng, Kun Chi, Guangyan Cai, Di Wu

**Affiliations:** Department of Nephrology, Chinese PLA General Hospital, Chinese PLA Institute of Nephrology, State Key Laboratory of Kidney Diseases, National Clinical Research Center for Kidney Diseases, Beijing Key Laboratory of Kidney Diseases, China

## Abstract

Kidney renal papillary renal cell carcinoma (KIRP) accounts for 10–15% of renal cell carcinoma (RCC). The need to find more therapeutic targets for KIRP is urgent because most targeted drugs have limited effects on advanced KIRP. Insulin-like growth factor (IGF) binding protein 5 (IGFBP5) is a secreted protein related to cell proliferation, cell adhesion, cell migration, the inflammatory response and fibrosis; these functions are independent of IGF. In our study, we determined the expression and functions of IGFBP5 with data from the database of The Cancer Genome Atlas (TCGA). We found that IGFBP5 is down regulated in KIRP kidney tissues compared to its expression in control tissues and that the expression of IGFBP5 is negatively related to patient survival. Bioinformatic analysis showed the probable processes and pathways involved in altered IGFBP5 expression, including blood vessel development, the cellular response to growth factor stimulus, the response to transforming growth factor *β* (TGF-*β*), and extracellular matrix organization. We proposed that VEGFA and TGF-*β* act as upstream regulatory factors of IGFBP5 and verified this in the Caki-2 cell line. Based on our results, we suggest that IGFBP5 might be a therapeutic target of KIRP.

## 1. Introduction

Renal cell carcinoma (RCC) is a common kind of malignant tumor originating from the epithelium of renal tubules. The most frequent forms of RCC are clear cell renal cell carcinoma (ccRCC), kidney renal papillary renal cell carcinoma (KIRP), and kidney renal chromophobe renal cell carcinoma (KICH). ccRCC accounts for 60–70% of RCC, and KIRP accounts for 10–15% of RCC. Treatment of advanced RCC rely on targeted drugs, such as sorafenib [1], which targets the RAF/MEK/ERK-induced signal transduction pathway and VEGFR, and sunitinib [2], which is a targeted receptor tyrosine kinase inhibitor. These targeted drugs have been approved as first-line drugs for metastatic RCC. However, most of these drugs are targeted on ccRCC but have limited effects on advanced KIRP. Because of the different mechanisms of KIRP and ccRCC and the low proportion of KIRP in RCC, KIRP patients have been excluded from large clinical trials of these targeted drugs [3], and research on KIRP progresses slowly. Although some KIRP patients can be diagnosed by ultrasonography and receive surgery at an early stage, a large number of advanced KIRP patients miss the opportunity due to the low efficiency of targeted drugs. Thus, the need to find more therapeutic targets in KIRP is urgent.

In this study, we found that insulin-like growth factor binding protein 5 (IGFBP5) is associated with KIRP patient survival and is a probable therapeutic target in KIRP. IGFBP5 is a secreted protein with a molecular weight of 30.57 kDa and it is an IGF-binding protein which is belonged to IGFBPs family. IGFBPs family is a group of proteins that are capable to bind IGF and have the two-way effects on IGF I and IGF II. The family consists of six identified proteins named IGFBP1 to IGFBP6. These proteins, in addition to being as the binding protein of IGF, have very important functions independent of IGF, especially in the progression of carcinoma. The main function of IGFBP5 is to bind circulating IGF and prolong its half-life [4]. Furthermore, an increasing number of studies have shown that IGFBP5 is related to cell proliferation, cell adhesion, cell migration, the inflammatory response and fibrosis independent of IGF [5–8]. This study focused on the relationship between IGFBP5 and KIRP determined from data from The Cancer Genome Atlas (TCGA) and describes the primary verification of this relationship.

## 2. Materials and Methods

### 2.1. Clinical Cohorts and RNA-Seq Data

Clinical cohort and RNA-seq data were downloaded from TCGA (http://www.tcga.org/). A total of 290 KIRP patients and 32 normal controls were included in the analysis. The clinical data included the patients' age, gender, race, neoplasm staging and survival time.

### 2.2. Analysis of RNA-Seq Data

Differential expression analysis between the normal controls and KIRP patients and Kaplan-Meier survival curve analysis were conducted with the Human Protein Atlas (https://www.proteinatlas.org), UALCAN analysis tools (http://ualcan.path.uab.edu/) [9] and SPASS 22.0. Bioinformatic analysis of the correlated genes included gene ontology (GO) and protein-protein interaction (PPI) analysis with Metascape analysis tools (http://metascape.org/) [10] and the Cbioportal for cancer genomics (http://www.cbioportal.org/) [11]. All these analysis tools are publicly available online.

### 2.3. Verification

#### 2.3.1. Tissue Sources

The expression of IGFBP5 in three pairs of human kidney tissues, including paracarcinoma and carcinoma tissues, was verified at the protein level with Western blotting and at the mRNA level with qPCR. The tissues were obtained from three KIRP patients who underwent surgery in the Urological Surgery unit of the Chinese PLA General Hospital. KIRP patient numbers are No. 101, No. 226, No. 246. This study was approved by the ethics committee of the Chinese PLA General Hospital (No. S2015-061-01) and carried out according to all the ethical standards of the institutional research committee and the Declaration of Helsinki.

#### 2.3.2. Cell Culture and Gene Silencing

The expression of IGFBP5, VEGFA and TGF-*β* was verified in the Caki-2 cancer cell line (ATCC, HTB-47). Cells were cultured in high-glucose Dulbecco's modified Eagle's medium (H-DMEM, Corning, 10-013-CVR) with 10% fetal bovine serum (FBS, Corning, 35-010-CV) and 1% penicillin-streptomycin (Corning, 30-002-CI) at 37°C in a humidified 5% CO_2 _environment.

VEGFA siRNA [12], TGF-*β* siRNA [13] and IGFBP5 siRNA [14] were used to inhibit the expression of VEGFA and TGF-*β*, respectively. The siRNA and primer sequences are shown in Table 1. Caki-2 cells were reseeded in 6-well plates before siRNA transfection. The cells were transfected with siRNA (2.5 *µ*g) by Lipofectamine 2000 reagent (10 *µ*l per well). The medium was changed to fresh complete medium after 6 h, and the cells were harvested with TRIzol reagent (Thermo Fisher Scientific, 15596026) after 24 hours.

#### 2.3.3. Cytokine Intervention

The cells were reseeded into 6-well plates and synchronized for 12 hours at 60% confluence. Then, the medium was replaced with H-DMEM with 2% FBS and VEGFA (5 ng/ml) or TGF-*β* (5 ng/ml), and cells were harvested with TRIzol after 48 hours.The cytokines used in the study were animal-free recombinant human VEGF165 (Peprotech, AF-100-20) and animal-free recombinant human TGF-*β* (Peprotech, AF-100-21C).

#### 2.3.4. qPCR Verification

A First Strand cDNA Synthesis Kit (New England Biolabs, E6560S) was used for cDNA synthesis with a standard protocol. First, the RNA sample (up to 1 *µ*g) was mixed with the d(T)_23_VN primer (2 *µ*l) and nuclease-free H_2_O (to a total volume of 8 *µ*l) in a sterile RNase-free microfuge tube. Second, the RNA/d(T)_23_VN sample was denatured for 5 minutes at 65°C, spun briefly and promptly placed on ice. Third, 10 *µ*l reaction mix (2×) and 2 *µ*l enzyme mix (10×) were added to the microfuge tube. Finally, the 20 *µ*l cDNA synthesis reaction was incubated at 42°C for one hour, and the enzyme was inactivated at 80°C for 5 minutes.

A SYBR Select Master Mix kit (Applied Biosystems, 4472908) was used for PCR with the following protocol. cDNA (1 *µ*l), 8 *µ*l nuclease-free H_2_O, 10 *µ*l reaction mix, and 1 *µ*l primer were added to 8-strip PCR tubes, and PCR was conducted through a standard procedure by an Applied Biosystems 7500 Real-Time PCR system.

#### 2.3.5. Western Blot Verification

Western blotting was used to detect the expression of IGFBP5 in kidney tissues from KIRP patients. Protein lysates were extracted from 50 mg each paracarcinoma and carcinoma tissue with cell lysis buffer (Beyotime, P0013B) by a tissue homogenizer. The protein concentration was detected by a BCA assay (Thermo Pierce, 23225), and proteins were denatured with LDS sample buffer (Invitrogen, NP0007). A total of 30 *μ*g protein was subjected to electrophoresis on SDS-PAGE gels and transferred onto nitrocellulose (NC) membranes (Pall BioTrace, 66485). The membranes were blocked with 5% milk, incubated with primary antibody overnight at 4°C and incubated with a secondary antibody after washing with TBST 3 times for 7 minutes each. Finally, proteins on the membranes were detected with a chemiluminescent reagent.

The following antibodies were used for Western blotting: primary antibodies against IGFBP5 (Proteintech, 55205-1-AP, rab) and GAPDH (CST, 2118, rab) and a goat anti-rab IgG-HRP secondary antibody (Beyotime, A0208). Proteins were detected with the BeyoECL Plus (Beyotime, P0018s) chemiluminescent reagent.

#### 2.3.6. Cell Proliferation Assay

The MTT assay (Beyotime, C0009) was used to determine cell proliferation according to the manufacturer's instructions. Cells were seeded onto 96-well plates and divided into normal control, IGFBP5 knockdown, IGFBP5 stimulation, VEGFA stimulation and TGF-*β* stimulation group. The MTT reagent was added to the medium at 0 h, 12 h, 24 h, and 48 h after intervention respectively and then incubated for 4 hours. The formazan precipitate was dissolved in solving reagent. The optical density was measured at a wavelength of 570 nm.

#### 2.3.7. Statistical Analysis of Verification

Experimental Data were presented as the mean ± standard deviation (SD). The two-tailed Student's *t*-test was used to analyze the difference of the experimental data between two groups, including qPCR, Western blot and cell proliferation. All calculations were performed with the Graphpad Prism 5.0 software. *p* < 0.05 was considered statistically significant.

## 3. Results

### 3.1. Clinical KIRP Patient Data

Clinical data from KIRP patients (Table 2) were downloaded from TCGA and supplemented with the Human Protein Atlas and UALCAN analysis tools. A total of 290 patients were included in the study, of which 214 were male and 76 were female. The ratio of males to females was approximately 3:1, the average age was 61.67 ± 11.89, and 58.6% (170/290) of the patients had stage I KIRP with a median survival time of 768.5 days. This is close to the natural population morbidity of KIRP, so the data represented the essential features of KIRP.

### 3.2. IGFBP5 Is Downregulated in KIRP

**Figure 1:**
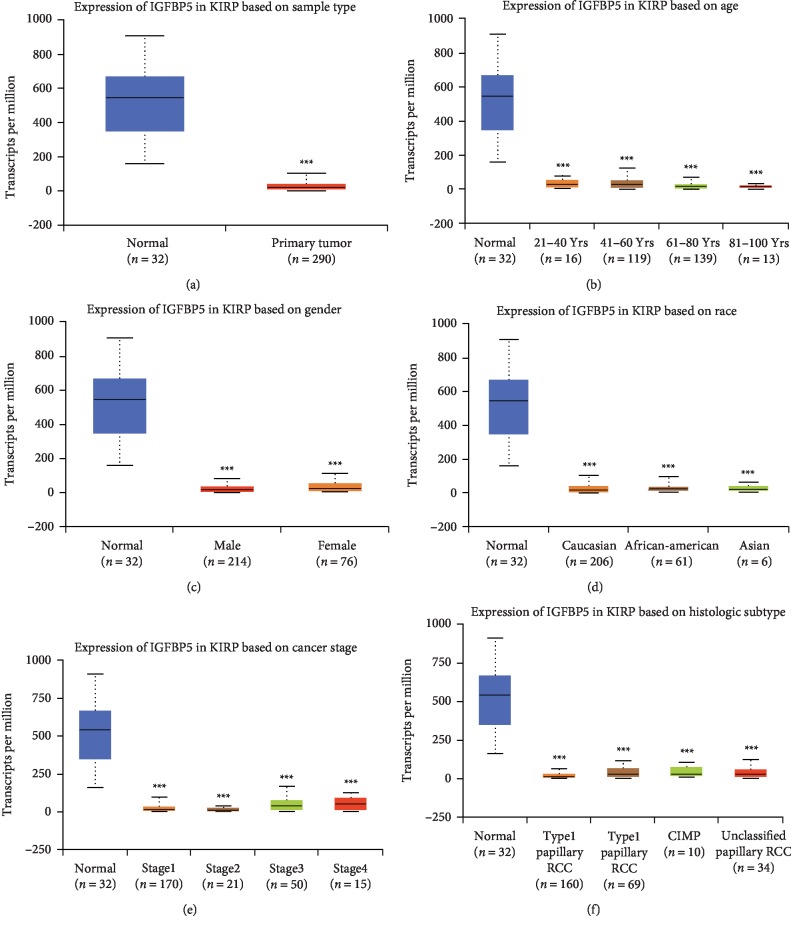
Expression of IGFBP5 in KIRP patient. (a) Expression of IGFBP5 in KIRP based on sample type. IGFBP5 is down expressed in KIRP compared with normal controls (*P* < 0.001). (b) Expression of IGFBP5 in KIRP based on age. The expression of IGFBP5 in each age subgroup has significance with normal group. (c) Expression of IGFBP5 in KIRP based on gender. The expression of IGFBP5 in each gender subgroup has significant with normal group, but there is no significance between male and female KIRP patients. (d) Expression of IGFBP5 in KIRP based on race. (e) Expression of IGFBP5 in KIRP based on cancer stage. (f) Expression of IGFBP5 in KIRP based on histologic subtype. KIRP = kidney renal papillary renal cell carcinoma.

IGFBP5 was significantly downregulated in KIRP patients compared with IGFBP5 levels in normal controls (Figure 1(a)). The transcript per million (TPM) level in normal controls and KIRP patients was 544.078 (351.367, 665.651) vs 18.742 (9.386, 35.083), respectively (*P* < 0.001) [data format: median (lower quartile, upper quartile)].

Then, we classified IGFBP5 expression in KIRP patients by patient gender, age, race, histologic subtype and cancer stage and found significant differences in IGFBP5 expression between the normal controls and all the subgroups (Figures 1(b)–1(f)). IGFBP5 expression levels were significantly different among the 41-60 year and 81–100 year age subgroups, and the 61–80 year and 81–100 year age subgroups (Figure 1(b)). IGFBP5 expression levels were significantly different among subgroups with KIRP at different stages, including stage I and stage II, stage II and stage III, and stage II and stage IV (Figure 1(e)), but we do not think these differences in IGFBP5 expression have clinical implications because the number of samples was limited, and the expression levels of IGFBP5 in different groups were very similar. A larger number of samples are needed to confirm the differences within subgroups of KIRP patients classified by age, gender, race, weight, subtype and stage. In conclusion, IGFBP5 is stably expressed at low levels in KIRP patients and therefore may be explored as a biomarker or target of KIRP.

### 3.3. IGFBP5 Is Related to the Survival of KIRP Patients

**Figure 2:**
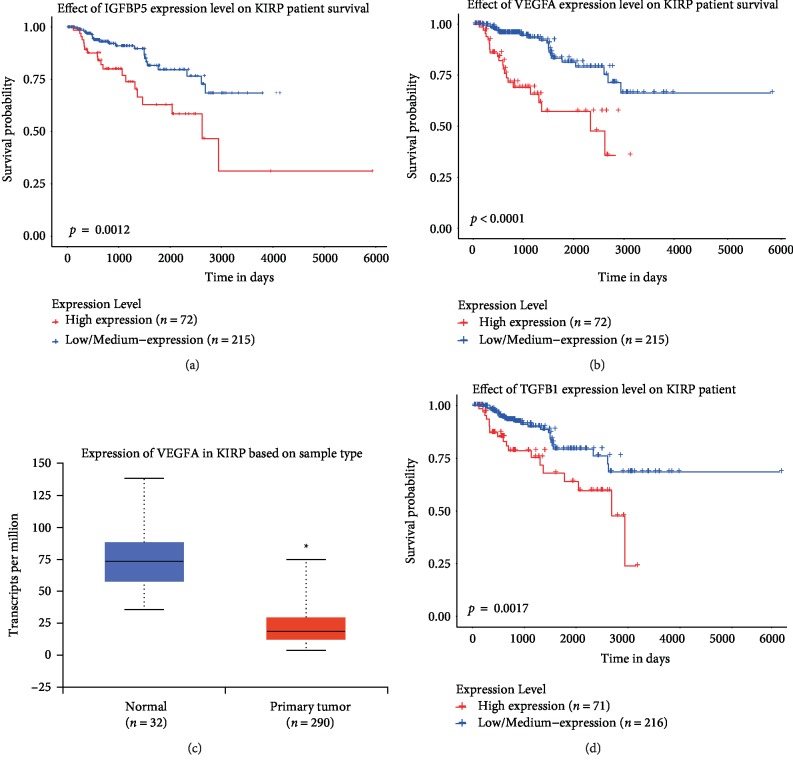
Survival curve of IGFBP5 and its related genes. (a) Kaplan-Meier survival curve of IGFBP5 for KIRP patients. The cut off value is 36.07. (b) Kaplan-Meier survival curve of VEGFA for KIRP patients. The cut off value is 6.34. (c) Expression of IGFBP5 in KIRP based on sample type. VEGFA is down expressed in KIRP compared with normal controls (*P* < 0.05) (d) Kaplan-Meier survival curve of TGF-*β* for KIRP patients. The cut off value is 6.34.

The survival rate was determined by Kaplan-Meier survival curve analysis of RNA-seq data from TCGA and clinical information. Not surprisingly, IGFBP5 is highly correlated with patient survival time, and as indicated by the Kaplan-Meier curve, low IGFBP5 expression was correlated with a longer survival time than high IGFBP5 expression (*P* < 0.001) (Figure 2(a)). The best cutoff value in Kaplan-Meier survival curve analysis was 36.07, and 72 patients were classified as exhibiting high IGFBP5 expression, while 215 patients were classified as exhibiting low IGFBP5 expression (data from three participants were lost). The median fragments per kilobase of transcript per million mapped reads (FPKM) in patients with low and high levels of IGFBP5 were 10.85 vs 56.1, respectively. Therefore, we suggest that low IGFBP5 expression might be a protective factor in KIRP patients.

### 3.4. Genes Positively Related to IGFBP5 in KIRP

#### 3.4.1. Basic Functions of IGFBP5 and Related Genes in KIRP

**Figure 3:**
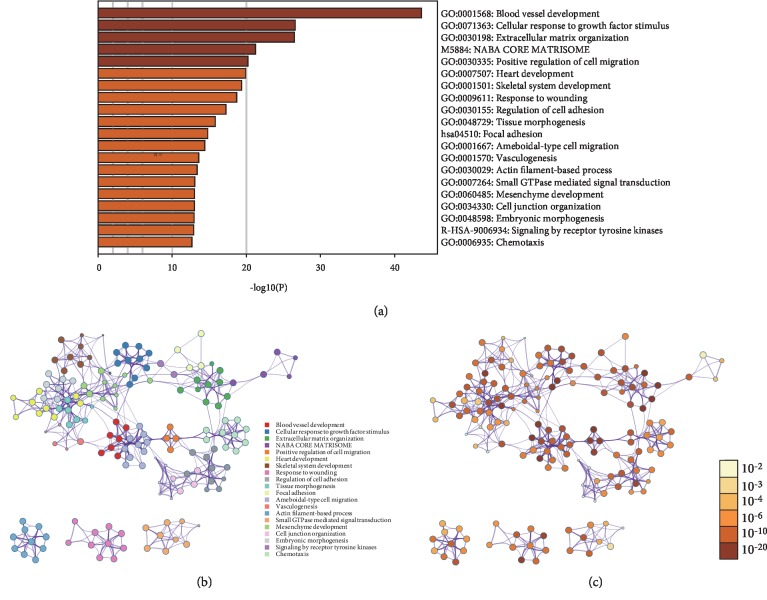
GO and PPI analysis of IGFBP5 related genes. (a) Main GO biological processes for IGFBP5 and its related genes analyzed by Metascape analysis tools. (b) The PPI networks determined by function cluster analysis. Different color represents for different function cluster. (c) The PPI networks determined by function cluster analysis. Different color represents for different *P* value. GO=gene oncology. PPI = protein-protein interaction.

To explore the possible function of IGFBP5 in the formation and progression of KIRP, we searched for genes related to IGFBP5 by UALCAN analysis tools and obtained 884 positively related genes with a Pearson correlation coefficient >0.3. A list of the top 34 genes with a Pearson correlation coefficient ≥0.7 is shown in Table 3. Then, we analyzed IGFBP5 and its related genes by Metascape analysis tools and found that the main GO biological processes for these genes were blood vessel development, cellular response to growth factor stimulus, response to transforming growth factor *β*, and extracellular matrix organization (Figure 3(a)). The PPI networks determined by function cluster analysis showed similar results (Figures 3(b) and 3(c)). These functions are well-known and active biological processes that take part in carcinoma. This indicates that the IGFBP5 protein is active in the progression of KIRP.

#### 3.4.2. IGFBP5 and Key Proteins in KIRP

To determine the complex mechanism by which one cytokine is involved in the progression of carcinoma and to explore the possible mechanism of IGFBP5 in KIRP, we identified two key proteins, VEGFA and TGF-*β*, which are highly related to IGFBP5 and actively participate in carcinoma.

VEGFA is a well-known cytokine that stimulates capillary proliferation in carcinoma. The Pearson correlation coefficient between VEGFA, which was in our list of genes positively correlated with IGFBP5, and IGFBP5 was high at 0.71. This suggests an interaction between IGFBP5 and VEGFA. GO and PPI analyses of the genes most positively related to IGFBP5 showed blood vessel development to be the most active biological process enriched in these genes. We compared the expression of VEGFA in normal control samples and samples from KIRP patients; the expression of VEGFA is significantly lower in KIRP patients than in normal controls (Figure 2(c)). Moreover, low VEGFA expression indicates a better KIRP prognosis (*P* < 0.0001) (Figure 2(b)). These trends are consistent with those observed for IGFBP5.

TGF-*β* is another canonical cytokine involved in carcinoma, and the Pearson correlation coefficient between TGF-*β* and IGFBP5 was 0.41 in our study. GO and PPI analyses of the genes most positively related to IGFBP5 showed the response to transforming growth factor *β* as the active biological process most enriched in these genes. Many studies have shown that excessive expression of TGF-*β* can promote the development and metastasis of carcinoma via stimulating the formation of peripheral blood vessels, inhibiting the immune system, forming the extracellular matrix, and promoting epithelial to mesenchymal transition (EMT). Based on these studies, we first examined the expression of TGF-*β* in normal samples and KIRP patients but found no significant difference. However, within the KIRP patients, patients with low TGF-*β* expression had a better prognosis than those with high TGF-*β* expression, as shown by survival curve analysis (*p* = 0.0017) (Figure 2(d)).

Thus, we hypothesized that VEGFA and TGF-*β* are key proteins that may regulate IGFBP5 expression.

#### 3.4.3. Verification

**Figure 4:**
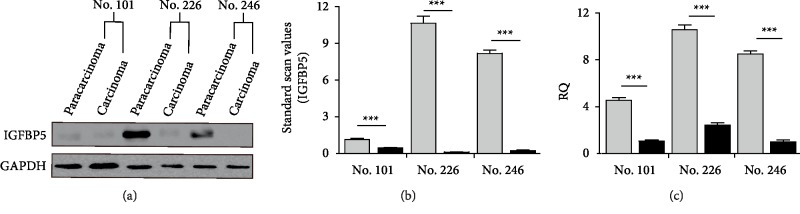
IGFBP5 expression in human kidney tissue. (a) IGFBP5 expression in carcinoma tissues compared with paracarcinoma tissues detected by Western blotting. (b) Semiquantitative analysis of IGFBP5 expression in (a) by ImageJ. (c) IGFBP5 expression in carcinoma tissues compared with paracarcinoma tissues detected by qPCR.

*(1) Verification in Kidney Tissue*: To verify the RNA-seq data, IGFBP5 expression in three pairs of paracarcinoma and carcinoma samples was determined by qPCR and Western blotting at the mRNA and protein levels, respectively. To verify differences in IGFBP5 protein expression, IGFBP5 was detected in 30 *µ*g of total protein lysate per sample by Western blotting, with GAPDH as a reference protein. Western blotting also showed significantly less IGFBP5 expression in carcinoma tissues than in paracarcinoma tissues (Figure 4(a)). Semiquantitative analysis by ImageJ showed IGFBP5 to be decreased by 2.5-fold, 100-fold, and 40-fold in samples No. 101, No. 226 and No. 246, respectively, compared to its expression in paracarcinoma tissue (Figure 4(b)). qPCR showed that the expression of IGFBP5 mRNA was significantly lower in carcinoma samples than in paracarcinoma samples among all three pairs, with an average difference in expression of approximately 5.22-fold (*p* < 0.001) (Figure 4(c)).

**Figure 5:**
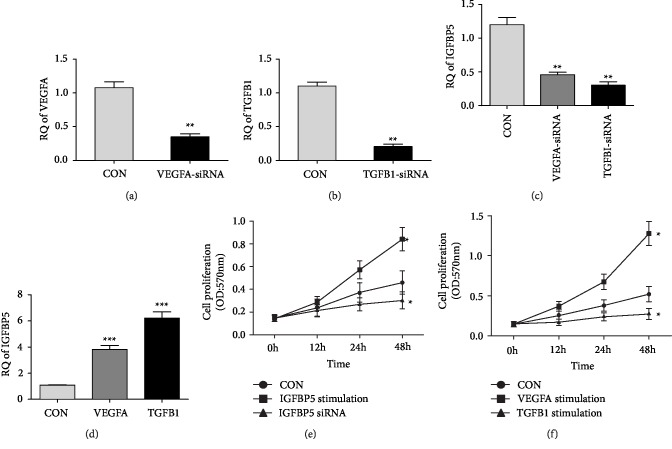
Expression of IGFBP5 in Caki-2 cell line. (a) Expression of VEGFA after inhibited by siRNA (*n* = 3). The fold change is 3.1 (*P* < 0.01). (b) Expression of TGF-*β* after inhibited by siRNA (*n* = 3). The fold change is 5.4 (*P* < 0.01). (c) Expression of IGFBP5 after VEGFA and TGF-*β* blocked by siRNA (*n* = 3). IGFBP5 was downregulated by 2.6-fold and 4.0 fold respectively (*P* < 0.01). (d) Expression of IGFBP5 after stimulated by VEGF_165 _and TGF-*β* (*n* = 3). IGFBP5 was upregulated by 2.8-fold and 6.2-fold respectively (*P* < 0.01). (e) Cell proliferation rate of Caki-2 detected by MTT assay (*n* = 3). The proliferation rate of IGFBP5 stimulation group is higher than normal control group (*P* < 0.05), and cell proliferation of IGFBP5 knockdown group is lower than normal control group (*P* < 0.05). (f) Cell proliferation rate of Caki-2 detected by MTT assay (*n* = 3). The proliferation rate of VEGF_165_ stimulation group is higher than normal control group (*P* < 0.05), and cell proliferation of TGF-*β* stimulation group is lower than normal control group (*P* < 0.05). (*n*  = 3) (∗, *p* < 0.05;∗∗, *p* < 0.01;∗∗∗, *p* < 0.001).

*(2) Verification in the Caki-2 Cell Line*: To demonstrate the relationship of IGFBP5 with VEGFA and TGF-*β* in tumor cells, we detected the expression of IGFBP5 in Caki-2 cells knocked down with VEGFA and TGF-*β*. VEGFA and TGF-*β* were successfully and efficiently inhibited by siRNA (Figures 5(a) and 5(b)). IGFBP5 was significantly downregulated after VEGFA and TGF-*β* were blocked by siRNA (Figure 5(c)). To confirm the interaction between IGFBP5 with VEGFA and TGF-*β*, we stimulated the Caki-2 cell line with animal-free recombinant human VEGF_165_ and TGF-*β*1. Not surprisingly, as shown by qPCR, IGFBP5 was upregulated (Figure 5(d)).

To explore the probable function of IGFBP5, we detected cell proliferation rate of Caki-2 by MTT assays. The results showed that cell proliferation levels of cells stimulated by IGFBP5 and VEGFA groups are higher than control group (*p* < 0.05) (Figures 5(e) and 5(f)). In contrast, the cell proliferation levels of cells knocked down of IGFBP5 is lower (*p* < 0.05) (Figure 5(e)). In addition, the cell proliferation level of cells stimulated by TGF-*β* is lower than normal control group (*p* < 0.05) (Figure 5(f)).

Based on all of these data, we confirmed that IGFBP5 is a protective factor of KIRP and that VEGFA and TGF-*β* are upstream regulators of IGFBP5.

## 4. Discussion

As IGF-binding proteins, the main function of members of the IGFBP family is to bind IGF in the circulation to prolong its half-life and regulate the bidirectional function of IGF [15]. In addition, the independent function of IGFBPs cannot be ignored. IGFBP5 has a unique function and mechanism of action and participates in several biological processes in carcinoma, such as cell proliferation, angiogenesis, cell migration and cell-cell adhesion, but these functions are bidirectional [6, 14, 16, 17].

In our study, IGFBP5 was downregulated in KIRP, and the survival curve showed a negative correlation between IGFBP5 expression and survival time. This suggests that the downregulation of IGFBP5 might be a protective factor of KIRP. What is the possible mechanism by which IGFBP5 participates in KIRP? GO and PPI analyses of genes most positively related to IGFBP5 showed active biological processes that including blood vessel development, cellular response to growth factor stimulus, response to transforming growth factor *β*, and extracellular matrix organization. In addition, the VEGFA and TGF-*β* genes were highly correlated with IGFBP5. Based on our data and those of previous studies, we analyzed VEGFA and TGF-*β* expression in KIRP.

VEGFA was downregulated in KIRP, and a low VEGFA level indicated a better prognosis than high VEGFA levels. VEGFA is a well-known cytokine that can activate tumor progression by stimulating blood vessel development [18]. However, VEGFA is downregulated in KIRP, and most large clinical studies of VEGF/VEGFR-targeted drugs in RCC patients showed unsatisfactory results in KIRP patients, unlike ccRCC patients [3]. A previous study showed that VEGFA is able to augment the expression of IGFBP5 in bovine aortic endothelial cells [19]. Thus, we hypothesized that VEGFA downregulation could downregulate the expression of IGFBP5 to inhibit tumorigenesis. Expression level of IGFBP5 in Caki-2 can be regulated by VEGFA siRNA and cytokine stimulation experiments and cell proliferation detection by MTT assays confirmed our hypothesis. Based on this evidence, we suggest that downregulated IGFBP5 expression induced by the downregulation of VEGFA might act as a protective factor in KIRP.

TGF-*β* is another canonical cytokine involved in carcinoma. Previous studies showed that TGF-*β* has the two-way effects on tumors. It was recognized as the suppress factor in carcinoma which can induce tumor cell apoptosis at first, but it's function of promoting tumor has been revealed by various studies [20]. Previous study showed that TGF-*β* is a key protein that induces tumor cell infiltration and metastasis via EMT, but has less effect on cell proliferation. EMT causes epithelial cells to lose their epithelial phenotype, including a loss of polarity and basal membrane rupture, and gain a mesenchymal phenotype, including migration and invasion, anti-apoptosis and enhanced extracellular matrix degradation [21]. MTT tests in our study also showed that cell proliferation level of Caki-2 cells in TGF-*β* stimulation group is lower than that in the normal control group. The results are in accord with previous studies.

However, few studies have investigated the interaction between IGFBP5 and TGF-*β* in carcinoma. A study targeting IGFBP5 and TGF-*β* in NMuMG cells revealed that IGFBP5 enhances epithelial cell adhesion and protects epithelial cells from TGF-*β*-induced mesenchymal invasion [22]. Several studies of idiopathic pulmonary fibrosis (IPF) [8] and rheumatoid arthritis [23] suggested a correlation between IGFBP5 and TGF-*β* in fibrosis and autoimmune disease, but the precise interaction between IGFBP5 and TGF-*β* is still unclear. GO and PPI network analyses showed that IGFBP5 functions in response to TGF-*β*, and expression and prognosis analysis showed the correlation between IGFBP5 and TGF-*β* in KIRP. TGF-*β* knockdown by siRNA and TGF-*β* cytokine stimulation experiments confirmed that IGFBP5 responds to TGF-*β* in the Caki-2 cell line. Based on our study, we propose that IGFBP5 is regulated by TGF-*β* and plays a role in tumor suppression.

In addition to VEGFA and TGF-*β*, TNF-α is another important cytokine correlated with IGFBP5, although TNF-α expression and survival prognosis were not significant different between KIRP patients and controls. There has been reported that IGFBP-5 inhibits TNF-α by competitively binding to TNFR1 [24, 25]. We hypothesize that downregulated IGFBP5 expression might release the binding domain of TNFR1 to restore apoptosis induced by TNF-α in cancer cells.

## 5. Conclusion

In conclusion, IGFBP5 is downregulated in KIRP kidney tissues, and low levels of IGFBP5 are correlated with a longer survival time in KIRP patients rather than high IGFBP5 levels. How IGFBP5 is involved in KIRP is unclear, but bioinformatics analysis has identified proteins, such as VEGFA, TGF-*β*, and TNF-α, and pathways, including blood vessel development, cell apoptosis, and the cellular response to transforming growth factor *β*, potentially central to the role of IGFBP5 in KIRP. Based on bioinformatics analysis and subsequent primary verification, we suggest that IGFBP5 is regulated by VEGFA and TGF-*β* and that IGFBP5 may play a protective role and be a potential drug target in KIRP. We are eager to determine the exact mechanism of IGFBP5 in more rigorous studies.

## Figures and Tables

**Table 1:** SiRNA and primer sequences.

Name	Sense/Forward	Antisense/Reverse
random siRNA	5′-UCCUCCGAACGUGUCACGUTT-3′	5′-ACGUGACACGUUCGGAGAATT-3′
VEGFA siRNA	5′-GGCAGAAUCAUCACGAAGUTT-3′	5′-ACUUCGUGAUGAUUCUGCCTT-3′
TGF-*β* siRNA	5′-GCAACAACGCCAUCUAUGATT-3′	5′-UCAUAGAUGGCGUUGUUGCTT-3′
IGFBP5 siRNA	5′-CGGGAGTCTCTCTCGATCCCTGTCTC-3′	5′-AGACAGGGAUCGAGAGAGACUCCCG-3′
VEGFA primer	5′-GGCAGAATCATCACGAAGTGGTG-3′	5′-GGGTCTCGATTGGATGGCAGTA-3′
TGF-*β* primer	5′-TTATTGAGCACCTTGGGCACT-3′	5′-TGCCATCTCAGAGTGTTGCT-3′
IGFBP5 primer	5′-CCCAATTGTGACCGCAAAGG-3′	5′-GGCAGCTTCATCCCGTACTT-3′
18S primer	5′-GTAACCCGTTGAACCCCATT-3′	5′-CCATCCAACGGTAGTAGCG-3′

**Table 2:** Clinical KIRP patient data.

	KIRP	
Total	290	

Gender	Male	214
	Female	76
	Unclear	0

Age	Years (mean, SD)	61.67, 11.89
	21–40	16
	41–60	119
	61–80	139
	81–100	13
	Unclear	3

Race	Caucasian	206
	African American	61
	Asian	6
	Unclear	17

Subtype	Type 1 papillary	160
	Type 2 papillary	69
	CIMP	10
	Unclassified	34
	Unclear	17

Stage	I	170
	II	21
	III	50
	IV	15
	V	0
	Unclear	29

Survival	Alive	241
	Dead	44
	Unclear	5

Survival time	Days	
	Median	768.5
	25%	421.3
	75%	1503
	Min	3
	Max	5925

**Table 3:** IGFBP5 related genes with a Pearson correlation coefficient ≥0.7.

Gene	Pearson correlation coefficient
TEK	0.79
PCDH12	0.78
PTPRB	0.77
KDR	0.77
ERG	0.77
SEMA3G	0.75
CLEC14A	0.75
LDB2	0.75
CD34	0.74
TMEM204	0.74
CDH13	0.74
NRIP2	0.74
UNC5B	0.74
CXorf36	0.73
TIE1	0.73
SLC7A1	0.73
COX4I2	0.73
MYCT1	0.73
ARHGEF15	0.72
ROBO4	0.72
CDH5	0.72
FLT1	0.72
EDNRA	0.72
MMRN2	0.71
RAMP3	0.71
INHA	0.71
VEGFA	0.71
GIMAP8	0.7
PODXL	0.7
TCF4	0.7
ELTD1	0.7
BCL6B	0.7
ENG	0.7
CYYR1	0.7

## Data Availability

The data sources used to support the findings of this study are included within the article.
